# Comparison of the effectiveness of different machine learning algorithms in predicting new fractures after PKP for osteoporotic vertebral compression fractures

**DOI:** 10.1186/s13018-023-03551-9

**Published:** 2023-01-23

**Authors:** Yiming Ma, Qi Lu, Feng Yuan, Hongliang Chen

**Affiliations:** 1 Department of Orthopaedic Surgery, Affiliated Hospital of Xuzhou Medical University, 99 Huaihai Road, Xuzhou, 221006 Jiangsu China; 2 Xuzhou Medical University, 209 Tongshan Road, Xuzhou, 221004 Jiangsu China

**Keywords:** Machine learning, Random forest, Logistic regression, Osteoporosis, Percutaneous kyphoplasty, New fracture

## Abstract

**Background:**

The use of machine learning has the potential to estimate the probability of a second classification event more accurately than traditional statistical methods, and few previous studies on predicting new fractures after osteoporotic vertebral compression fractures (OVCFs) have focussed on this point. The aim of this study was to explore whether several different machine learning models could produce better predictions than logistic regression models and to select an optimal model.

**Methods:**

A retrospective analysis of 529 patients who underwent percutaneous kyphoplasty (PKP) for OVCFs at our institution between June 2017 and June 2020 was performed. The patient data were used to create machine learning (including decision trees (DT), random forests (RF), support vector machines (SVM), gradient boosting machines (GBM), neural networks (NNET), and regularized discriminant analysis (RDA)) and logistic regression models (LR) to estimate the probability of new fractures occurring after surgery. The dataset was divided into a training set (75%) and a test set (25%), and machine learning models were built in the training set after ten cross-validations, after which each model was evaluated in the test set, and model performance was assessed by comparing the area under the curve (AUC) of each model.

**Results:**

Among the six machine learning algorithms, except that the AUC of DT [0.775 (95% CI 0.728–0.822)] was lower than that of LR [0.831 (95% CI 0.783–0.878)], RA [0.953 (95% CI 0.927–0.980)], GBM [0.941 (95% CI 0.911–0.971)], SVM [0.869 (95% CI 0.827–0.910), NNET [0.869 (95% CI 0.826–0.912)], and RDA [0.890 (95% CI 0.851–0.929)] were all better than LR.

**Conclusions:**

For prediction of the probability of new fracture after PKP, machine learning algorithms outperformed logistic regression, with random forest having the strongest predictive power.

**Graphical Abstract:**

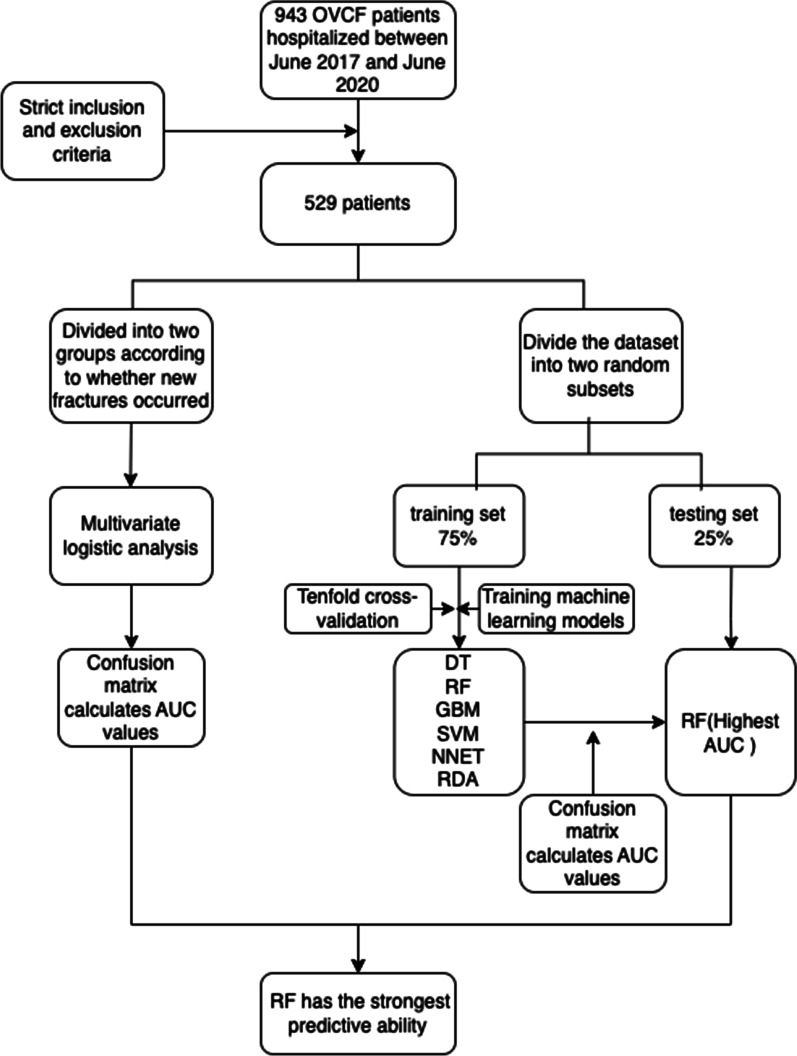

## Highlights

1. Machine learning significantly outperformed logistic regression models in predicting new fractures after osteoporotic vertebral compression fractures.
2. The random forest model performs best among these machine learning models.

## Introduction

One of the serious consequences of osteoporosis is osteoporotic vertebral compression fracture (OVCF), which affects more than 700,000 Americans every year [1]. By 2025, > 3 million osteoporotic fractures and $25 billion in related health care costs will occur in the USA [2]. OVCFs cause persistent back pain, kyphosis, and limited motion [3]. In 1999, Mark Reiley improved percutaneous vertebroplasty (PVP) and created percutaneous kyphoplasty (PKP) [4], which restores vertebral height and reduces pain by injecting cement into the fractured vertebral body in a minimally invasive way; thus, it has become the preferred treatment for OVCFs because of its ability to provide rapid relief and shorten recovery time [5].

Despite these advantages, there are many complications after PKP, among which new vertebral compression fracture (NVCF) is one of the most common complications, with an incidence of up to 50% [6, 7]. There are various statistical methods for estimating the probability of occurrence of binary events such as NVCF, the most commonly used being logistic regression (LR). With the rapid development in the field of artificial intelligence, machine learning (ML) is increasingly used in the medical field [8, 9], in particular, when using large datasets for prediction [10, 11]. However, it is unclear whether ML methods can provide better predictive power than traditional logistic regression algorithms. The purpose of this study was to investigate whether there is any difference in the probabilistic predictive ability of machine learning and logistic regression methods for predicting new fractures after OVCF surgery. We used six different machine learning methods to analyse our data: random forest (RF), gradient boosting machine (GBM), decision tree (DT), support vector machine (SVM), neural networks (NNET), and regularized discriminant analysis (RDA). The algorithm with the highest AUC was selected as the optimal algorithm.

## Materials and methods

### Inclusion and exclusion criteria

The data of 529 patients with OVCFs who were treated with PKP and completed at least 2 years of follow-up after attending our hospital from January 2017 to June 2020 were analysed. The inclusion criteria were as follows: (1) age ≥ 60 years; (2) high signal in T2 and low signal on T1 of the fractured vertebrae confirmed by preoperative magnetic resonance imaging; (3) bone mineral density *T* value ≤ − 2.5 measured by dual-energy X-ray; (4) follow-up time ≥ 2 year; (5) complete preoperative and postoperative clinical and imaging data of the patients; and (6) all patients completed the follow-up. The exclusion criteria were as follows: (1) symptomatic low back pain from other causes (disc herniation, slipped vertebrae, lumbar isthmus fracture, fall from height, etc.); (2) infectious diseases; (3) malignant tumours of the spine; (4) paralysis or loss of voluntary mobility; (5) unwillingness to complete follow-up; and (6) pathological fractures, posterior column fractures, and neurological symptoms. In addition, even if new vertebral fractures occurred multiple times during postoperative follow-up, only the first occurrence was included in the analysis.

### Observation factors

Preoperatively, according to the morphology of the fractured vertebrae based on imaging data, fractures involving the anterior column causing compression of the anterior column were classified as wedge-shaped fractures. When they were fractures that involved the middle column caused compression of the middle column, while the compression of the anterior and posterior column were not obvious, they were classified as biconcave fractures. When the anterior, middle, and posterior columns were all compressed, this type of fracture was classified as compression fracture. Fractures occurring in the T11-L2 vertebral body were defined as thoracolumbar fractures. The vertebral height recovery rate was calculated as (postoperative vertebral height − preoperative vertebral height)/normal vertebral height * 100%, where normal vertebral height was defined as (upper vertebral height + lower vertebral height)/2.

Postoperative frontal and lateral spine radiographs were taken in all patients to measure the segmental kyphosis of the operated segment. The segmental kyphosis angle was defined as the angle between the lower endplate of the upper vertebral body of the fractured vertebra and the upper endplate of the lower vertebral body.

Unilateral or bilateral distribution was defined according to whether the cement crosses the midline in the postoperative orthopantomogram of the spine; if the bilateral cement masses are discontinuous, the distribution is bilaterally separated. If vice versa, the distribution is bilaterally fused.

### Statistical methods

Continuous variables are expressed as the mean ± standard deviation ($\overline{x }$±s), and categorical variables are expressed as ratios [*n* (%)]. The data were randomly divided into a training set (75%) and a validation set (25%). Logistic regression was applied to analyse the independent risk factors for new postoperative fractures in PKP and to calculate the odds ratio (OR) and 95% confidence interval (95% CI). OR > 1 indicated that the variable was a positive risk factor for outcome, while OR < 1 indicated that the variable was a negative risk factor for outcome. *P* < 0.05 was considered statistically significant. Statistical analysis and modelling were performed using SPSS software (Version 20.0, IBM Corporation, Chicago, USA) and R Studio software (Version 25.0, R Foundation for Statistics Computing, Vienna, Austria). Various packages were used to train the models and plot the corresponding graphs, and the caret package was applied to train and validate the machine learning models.

**Fig. 1:**
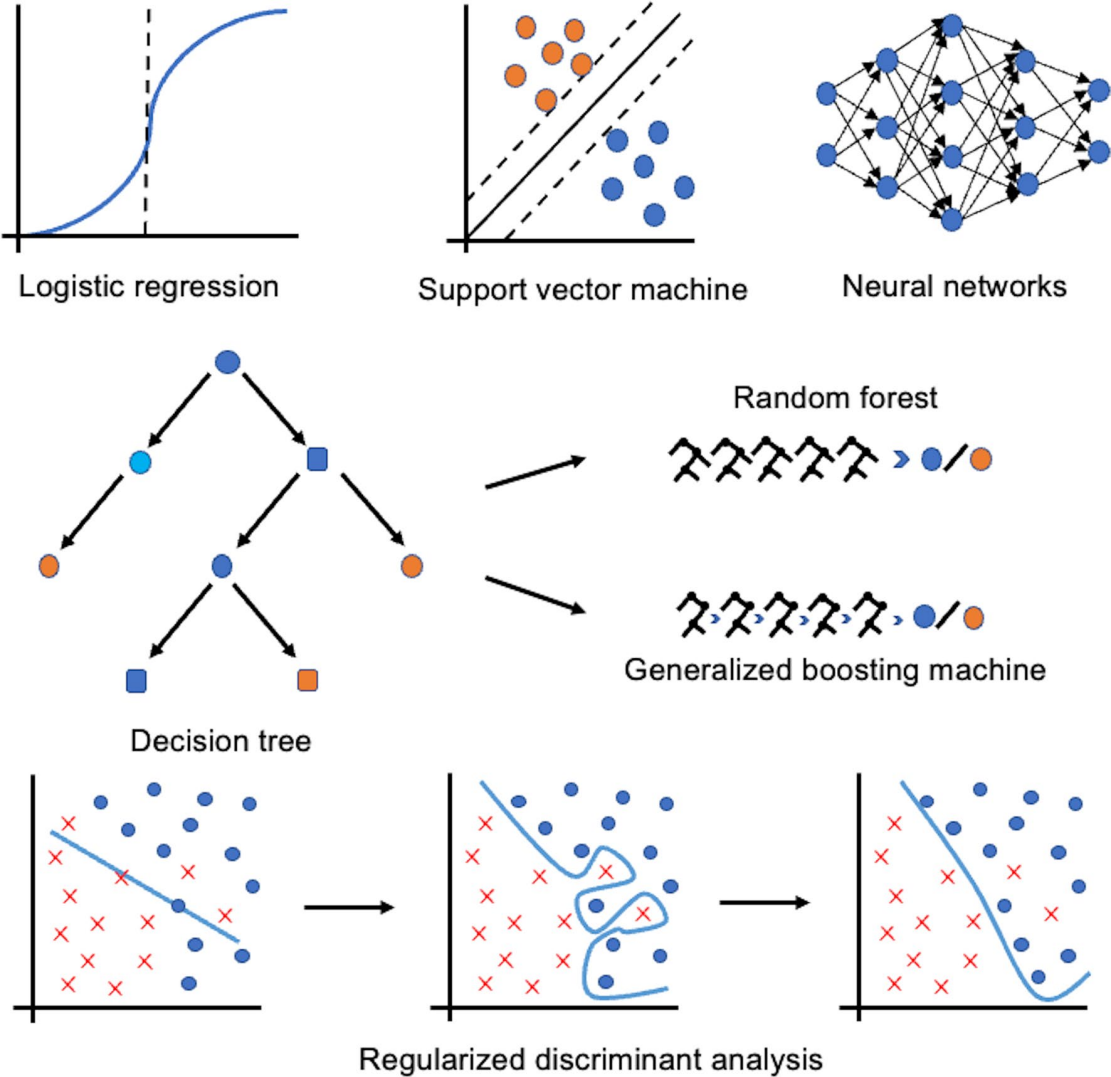
Classification algorithm. Logistic regression was used to calculate the probability of a binary ending event, fitting an S-shaped probability curve. Support vector machine is a binary classification model based on the principle of finding the hyperplane in a three-dimensional scatter plot that divides the dataset into two categories for smaller datasets. Neural networks are complex interconnected regression layers, such as biological neural networks in the human brain. Neural networks benefit from a large amount of data. Decision trees (e.g. random forests and gradient boosting machines, where random forests are algorithms that integrate multiple decision trees; gradient boosters are algorithms that iterate through multiple decision trees to improve predictive power) use a flowchart-like structure for decision-making that is easy to understand and visualize. The data points are split into similar categories (each "branch in the tree", so-called splitting points) at a given time. Regularized discriminant analysis can reduce the dimensionality of a binary ending dataset with many features and avoid overfitting to achieve sample balancing

As a complement to the logistic regression, six machine learning models are fitted: decision tree (DT), random forest (RF), gradient boosting machine (GBM), support vector machine (SVM), neural network (NNET) and regularized discriminant analysis (RDA). Random forest and gradient boosting machine are based on decision tree, and decision tree as a weak prediction model is usually implemented by optimizing the objective or voting. However, due to the limited capability of the algorithm, the prediction results are usually affected by the training set used to construct the model. Although the problem of overfitting the model to the training dataset can be solved by pruning, the prediction capability is relatively weak for a single tree model. Random forest avoids the phenomenon of data overfitting and can balance the error for unbalanced datasets. The algorithm is highly resistant to disturbances, and the model does not fail even when there are many missing samples. The principle of the gradient boosting machine is to use an additive model with a forward distribution algorithm and, based on this, a boosting method with a decision tree as the basis function, which is simply an additive model of many decision tree models. A support vector machine is built as a hyperplane with two hyperplanes parallel to each other on either side of the hyperplane that separates the data, thus dividing the patients into groups with the same outcome. The greater the distance or gap between the parallel hyperplanes, the smaller the total error of the classifier. Neural networks are derived by inputting multiple nonlinear models and a weighted interconnection between different models (the weighting process is performed in the hidden layer), resulting in an output model. Regularized discriminant analysis is an effective algorithm for datasets with many features to avoid underfitting and overfitting of the model and to equalize the sample (Fig. 1).

The area under the curve (AUC) of the receiver operating characteristic curve (ROC) was used as the main indicator to evaluate the predictive ability of the model, ranging from 0 to 1, with higher values indicating better predictive performance. The performance of the model was tested using tenfold cross-validation.

## Results

### Baseline data

**Fig. 2:**
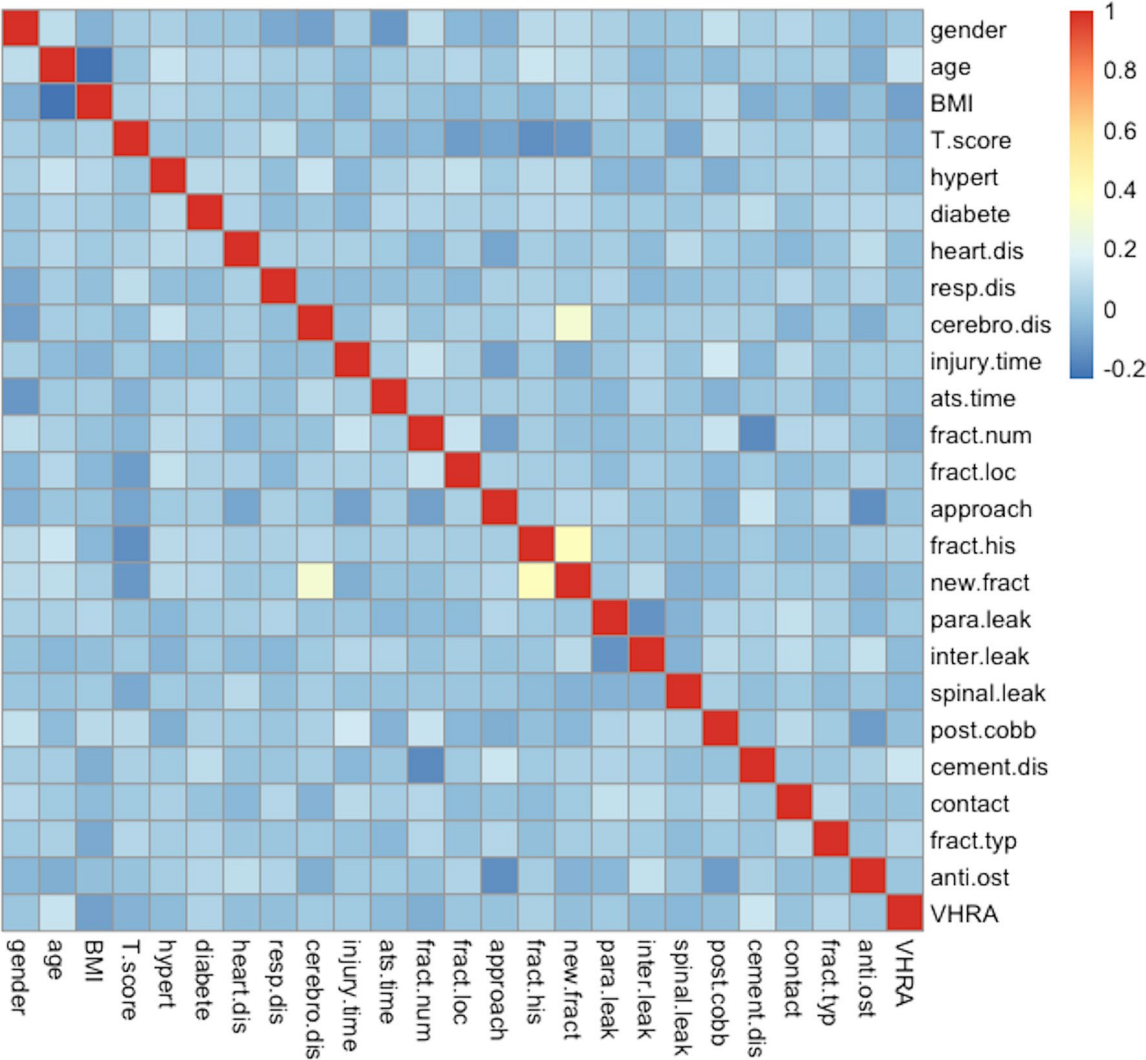
Heatmap. Each square indicates the correlation between the factors in that row and column, and the colour is used to indicate the amount of correlation. Factors near positive colours are high expressions and positive correlations, while factors near negative colours are low expressions and negative correlations. Abbreviation: hypert (hypertension); Heart.dis (heart diseases); resp.dis (respiratory diseases); cerebro.dis (cerebrovascular disease); ats.time (time from admission to surgery); fract.num (number of fractured vertebral); fract.loc (fracture vertebral location); para.leak (paravertebral leakage); inter.leak (intervertebral leakage); spinal.leak (spinal leakage); post.cobb (postoperation cobb); cement.dis (cement distribution); fract.typ (fracture type); anti.ost (anti-osteoporosis); VHRA (vertebral height recovery rate)

The mean age of the 529 patients was 71.185 ± 10.012 years, and a total of 56 (10.6%) had NVCF after surgery. The baseline data are shown in Table 1. BMI was included in this study as a better indicator of obesity, and only BMI was included without considering height or weight. Based on the collected patient data, a heatmap was made to show the relationship between the variables. The heatmap shows that cement intervertebral leakage and previous fracture history were most correlated with outcome (Fig. 2).

**Table 1:** Baseline characteristics [*n* (%), $\overline{x }$±s]

Characteristics	
Patients number	529
Age	71.185 ± 10.012
Gender (Male/Female)	135 (25.5)/394 (74.5)
BMI (kg/m^2^)	23.394 ± 3.299
BMD	− 2.811 ± 0.270
Hypertension	200 (37.8)/329 (62.2)
Diabetes	45 (8.5)/484 (91.5)
Heart disease	28 (5.3)/501 (94.7)
Respiratory system disease	7 (1.3)/522 (98.7)
Cerebrovascular disease	21 (4.0)/508 (96.0)
Injury time(days)	26.718 ± 67.426
Time from admission to surgery (days)	3.010 ± 2.552
Number of fractured vertebral	1.327 ± 0.693
Fracture vertebral location	
Thoracic/thoracolumbar/lumbar	72 (13.6)/347 (65.6)/110 (20.8)
Approach	
Unilateral/bilateral	147 (27.8)/382 (72.2)
Fracture history(Y/N)	90 (17.0)/439 (83.0)
New fracture(Y/N)	56 (10.6)/473 (89.4)
Paravertebral leakage	74 (14)/455 (86)
Intervertebral leakage	63 (11.9)/466 (88.1)
Spinal leakage	12 (2.3)/517 (97.7)
Postoperative Cobb	12.508 ± 8.293
Cement distribution	
Unilateral/bilateral fusion/ bilateral separation	119 (22.5)/285 (53.9)/125 (23.6)
Cement-endplate contact (Y/N)	417 (78.8)/417 (21.2)
Fracture type	
Wedge/biconcave/compression	208 (39.3)/264 (49.9)/57 (10.8)
Anti-osteoporosis(Y/N)	429 (81.1)/429 (18.9)
Vertebral height recovery rate	14.393 ± 13.441

### Multivariate logistic regression analysis

In the multivariate logistic regression analysis (Table 2), the results showed that sex [OR = 2.621, 95% CI (1.030–6.673), *P* = 0.043], cerebrovascular disease [OR = 28.522, 95% CI (8.749–92.989), *P* = 0.000], fracture history [OR = 12.298, 95% CI (6.250–24.199), *P* = 0.000], and intervertebral leakage of bone cement [OR = 2.501, 95% CI (1.029–6.082), *P* = 0.043] were independent predictors of NVCF.

**Table 2:** Univariate and multivariate analysis

Variates	Odd ratio	Lower	Upper	*P*
Gender	2.621	1.030	6.673	0.043
Cerebrovascular disease	28.522	8.749	92.989	0.000
Injury time	0.985	0.969	1.001	0.065
Fracture history	12.298	6.250	24.199	0.000
Intervertebral leakage	2.501	1.029	6.082	0.043

‘Lower’ and ‘upper’ represent the bounds of the 95% confidence interval

### Variable importance ranking

**Fig. 3:**
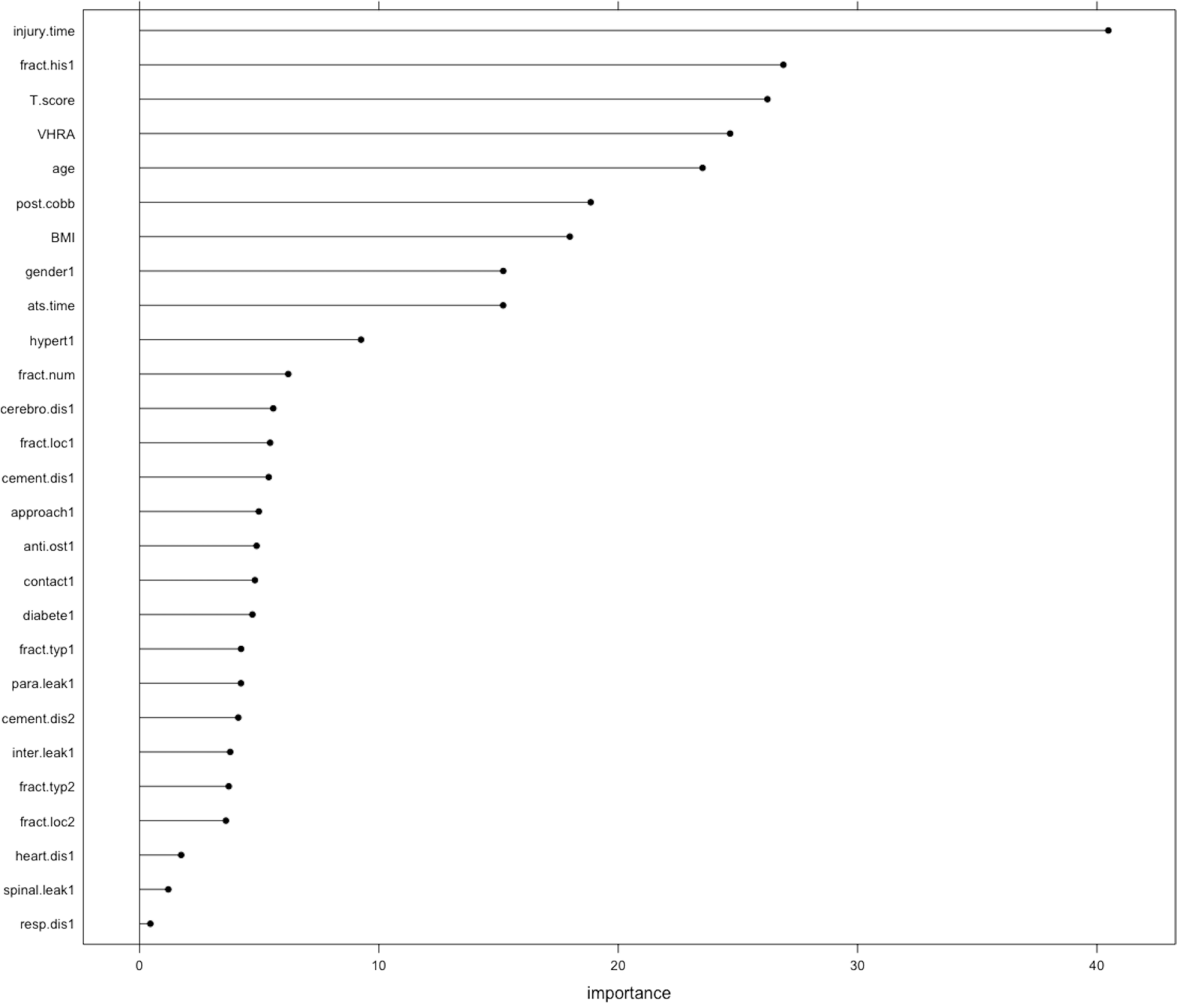
Variable importance distribution in the random forest model. Time of injury, history of previous fracture, bone density, rate of vertebral height recovery, and age were ranked in order of importance for new fractures after surgery. Other factors in the random forest model were not important enough for the effect of new fractures after surgery

The random forest-based variables ranked in order of importance were time of injury, fracture history, BMD, vertebral height recovery rate, age, postoperative Cobb angle, BMI and female sex. Time from admission to surgery, hypertension, number of fractured vertebrae, cerebrovascular disease, cement bilateral fusion distribution, thoracolumbar segment fracture, cement bilateral fusion distribution, bilateral approach, anti-osteoporosis treatment, cement-endplate contact, diabetes, wedge fracture, paravertebral leakage, cement bilateral separation distribution, cement intervertebral leakage, compression fracture, lumbar fracture, heart disease, cement spinal leakage, and respiratory disease performed poorly and in descending order (Fig. 3).

### Performance of machine learning algorithms

To compare the prediction performance of different algorithms, our study used the AUC from a tenfold cross-validation and validated it on a test set as the main index to evaluate the model performance. Among the six machine learning models, only the decision tree [0.842 (0.796–0.888)] had a lower AUC value than logistic regression [0.898 (0.860–0.936)], and the random forest model predicted NVCF best with an AUC of 0.948 (95% CI 0.920–0.977). The results are shown in Table 3.

**Table 3:** Evaluation of prediction performance of each model

Models	AUC (95% CI)	Kappa	Sensitivity	Specificity
Logistic regression	0.898 (0.860–0.936)	0.794	0.831	0.965
Random forest	0.940 (0.910–0.970)	0.880	0.966	0.913
Gradient boosting machine	0.910 (0.873–0.947)	0.820	0.898	0.922
Decision tree	0.842 (0.796–0.888)	0.683	0.779	0.904
Support vector machine	0.902 (0.864–0.940)	0.803	0.856	0.948
Neural network	0.923 (0.889–0.957)	0.846	0.907	0.939
Regularized discriminant analysis	0.915 (0.879–0.950)	0.828	0.881	0.947

The Kappa values are based on the confusion matrix used for consistency testing, with results in the range of 0–0.2 for SLIGHT, 0.21–0.4 for FAIR, 0.41–0.60 for MODERATE, 0.61–0.80 for SUBSTANTIAL, and 0.81–1 for ALMOST PERFECT. Sensitivity is the percentage of true positive samples among actual positive samples, and specificity is the percentage of true negative samples among actual negative samples

## Discussion

PKP has led to symptom reduction, functional recovery and improved quality of life in OVCF patients. However, there are still postoperative complications that cannot be ignored, and NVCF is one of the most common complications. It is increasingly important to better anticipate and reduce the occurrence of complications in advance. Therefore, accurate predictive models are needed to help clinicians and patients share decisions and understand the risk of postoperative complications. With advances in the field of artificial intelligence, machine learning is often able to perform better than traditional linear models [12, 13].

Machine learning uses computer algorithms to learn complex relationships or patterns between data from large amounts of data, recognizes the data by training existing algorithms to perform many operations, and iteratively changes the algorithms to achieve optimal performance, resulting in models that relate multiple feature variables to the target variable [14]. Specifically, supervised machine learning identifies relationships between input and output data (i.e. the computer learns from patient data) to produce outcome predictions based on the input data [15]. Clinicians can use this AI-based strategy to help them choose more rational treatment options. ML has the advantage of being highly capable, objective and reproducible when dealing with large datasets with reliable results [16]. It also has the potential to improve the accuracy of early diagnosis, determine disease progression, and improve the ability to predict patient outcomes, such as the risk of complications [17]. These advantages can facilitate the sharing of information for decision-making between clinicians and patients and keep track of disease progression [18]. Machine learning improves the efficacy of predicting clinical outcome metrics by constructing different algorithms for evaluation and comparison. Machine learning algorithms include support vector machines, random forests, gradient boosting machines, neural networks, and deep learning, in addition to traditional logistic regression and decision trees, which have been extended on this basis [19]. With the availability of computers and large clinical datasets, machine learning as a form of artificial intelligence has started to be used in clinical settings to assist in medical decision-making.

PKP has become a common treatment for OVCFs, but the incidence of postoperative NVCFs can be as high as 50% [20]. In a random forest-based ranking of the importance of variables, patients with a shorter time to fracture were at greater risk of postoperative NVCF (Fig. 3), and patients with a longer time to injury had a greater increase in bone formation markers than bone resorption markers [21]. Progressive degeneration may increase the lumbar BMD over time compared with lower lumbar BMD values and a longer duration of back pain in patients hospitalized for surgery within a short period of time after symptom onset. Fresher fractures may cause oedema or haematoma around and within the fractured vertebra and may lead to prolonged back pain and poor functional recovery. A review [22] showed that for patients with OVCF within 6 weeks, PKP did not demonstrate a significant benefit compared to placebo. A meta-analysis by Buchbinder et al. [23] reported no significant differences in pain, disability, quality of life, or outcomes with PKP compared to sham surgery. Therefore, from a clinical perspective, the treatment goals for patients with fresh fractures should focus on strategies that first improve functional recovery, relieve back pain, and increase lumbar spine BMD values.

In this study, previous fracture history [OR = 12.298, 95% CI (6.250–24.199), P = 0.000] was the most effective predictor of postoperative fracture risk. Many patients with clinically asymptomatic previous fractures are found to have a history of previous vertebral fractures on imaging at the next visit to the hospital when the fracture arises, and therefore, patients do not recognize the danger of osteoporosis until symptoms develop. It has been reported [24] that individuals with a history of clinically diagnosed fractures or radiographic evidence of vertebral fracture patterns are at increased risk for hip, spine, and other fractures. It was also reported that approximately half of women with fracture patterns did not report having back pain, and approximately two-thirds had no previous clinical diagnosis of fracture. A study by Torgerson et al. [25] examined the association between multiple fractures and secondary fractures, with a 5.9-fold increased risk of secondary fractures in women with two or more fractures. This suggests that the risk of a new fracture following the presence of multiple prior fractures is greatly increased. Therefore, patients with a history of fractures should undergo further evaluation for osteoporosis and fracture risk.

The degree of osteoporosis is a major risk factor for the development of postoperative NVCF, and bone mineral density, an index to assess the mineral content of bone, is commonly used to diagnose osteoporosis [26]. Ning et al. [27] found that by including 921 cases with low *T* values, bone trabeculae that were denser became sparse, leading to reduced bone support and toughness, an increased risk of fracture postoperatively and a greater risk of NVCF. It has been suggested that the progression of osteoporosis is associated with the development of postoperative NVCFs [28] and that anti-osteoporosis treatment may slow the progression of osteoporosis and prevent the development of NVCFs [27–29]. A 3-year follow-up study by Bawa et al. [30] showed that effective anti-osteoporosis treatment significantly reduced the incidence of postoperative VCFs. Multifactorial analysis showed that ineffective anti-osteoporosis treatment was a significant risk factor for NVCFs after PKP surgery. Therefore, anti-osteoporosis therapy should be used as a routine treatment after PKP in patients with OVCFs to reduce the incidence of refracture.

Leakage of bone cement through the ruptured endplate into the intervertebral disc causes changes in the surrounding vertebral body stresses and changes in the stiffness of the injured vertebral body due to reinforcement of the injured vertebral body but has a limited effect on the adjacent vertebral body after cushioning by the disc. However, when bone cement leaks into the intervertebral disc, it can increase the stress on the endplate of the adjacent vertebral body, and this alteration may increase the risk of new vertebral fractures [31]. A study by Nieuwenhuijse et al. [32] found a significant association between bone cement leakage into the disc and the occurrence of postoperative NVCF. Multifactorial analysis in this study showed that leakage of bone cement into the intervertebral disc [OR = 2.501, 95% CI (1.029–6.082), P = 0.043] was a risk factor positively associated with new fractures. In addition, the heat generated by the bone cement leaking into the intervertebral disc may cause some damage to the disc, which may also be a major contributor to accelerated disc degeneration [33].

Oestrogen can directly affect bone metabolism by regulating cellular physiological functions. The decrease in oestrogen levels in postmenopausal women inevitably leads to the weakening of its inhibitory effect on osteoclasts, an increase in the number of osteoclasts, a decrease in apoptosis, and the prolongation of lifespan, which enhances bone resorption and promotes the progression of osteoporosis. Although osteoblast-mediated bone formation was also increased, it was not sufficient to compensate for excessive bone resorption. Active and unbalanced bone remodelling leads to thinning or fracture of trabecular bone, increased cortical bone porosity leads to decreased bone strength, and decreased oestrogen reduces bone sensitivity to mechanical stimulation, resulting in bone exhibiting pathological changes such as disuse bone loss. [34] A multicentre large-sample cohort study on the prevalence of osteoporosis in Chinese individuals by Zeng et al. [35] found that the number of women suffering from osteoporosis is much greater than that of men. In the USA, approximately 1 in 2 white women or 1 in 5 men will experience an osteoporosis-related fracture in their lifetime [36]. However, in a large cross-sectional study by Wang et al. 18, it was found that in China, 5.0% of men and 20.6% of women aged 40 or older had osteoporosis, and 10.5% of men and 9.7% of men aged 40 or older had vertebral fractures. The similar prevalence of vertebral fractures in men and women suggests that we should also pay attention to the prevention and treatment of osteoporosis in men.

Multivariate analysis showed that the presence of cerebrovascular disease [OR = 28.522, 95% CI (8.749–92.989), *P* = 0.000] was associated with a higher risk of postoperative NVCF. A study by Tanislav et al. [37] showed that the occurrence of stroke as well as transient cerebral ischaemia was positively associated with fracture. Various adverse outcomes, such as depression, pain and reduced quality of life following stroke occurrence, lead to a higher risk of falls and fractures [38]. A large cohort study by Wang et al. [39] found that patients had a risk of fracture of more than 8% 5 years after stroke occurrence and that stroke was significantly associated with fracture risk. Stroke in certain vascular regions of the brainstem can lead to impaired body balance and increased risk of falls [40]. In addition, impairment of visual, motor, sensory or cognitive function after the onset of cerebrovascular disease may also lead to fall-related injuries [41]. Within 2 years after stroke, 60.7% of fallers experienced a second or multiple falls, and 23.4% of patients had a fracture [42]. In addition to falls, the accelerated decrease in bone mineral density after stroke may lead to fractures in stroke patients [43]. Poststroke muscle weakness leads to limited weight bearing and reduced activity of the limb, which results in reduced bone mass. In addition, malnutrition, reduced sun exposure, and vitamin D deficiency can exacerbate bone loss in stroke survivors. Common stroke treatments, such as oral anticoagulants, can also increase the risk of osteoporosis and fracture [44]. Therefore, effective measures should be taken for skeletal health screening and fracture prevention in patients with cerebrovascular disease.

A comparison of multiple machine learning algorithms showed that random forests performed best in predicting the risk of postoperative NVCF. Our study has several advantages. First, few studies have examined which of the logistic regression and machine learning algorithms is better in predicting the probability of NVCF after PKP. Furthermore, our model shows superior predictive ability compared to other models. However, this study also has some limitations. First, the nature of retrospective studies can lead to subjective and selection bias. Second, the sample size included in the single-centre study is still not large enough. Third, because most clinicians have a low level of understanding of techniques such as machine learning, this may limit the dissemination and application of the study results. Finally, single-centre studies may limit the sample selection and its applicability to other regions, so we need further external validation with multicentre data. In this paper, we found that the random forest algorithm has good performance in predicting bone cement leakage after orthopaedic surgery, and at the same time, it has comparable accuracy and ease of use. In the future, we will collaborate with more countries and institutions to include patient samples from different countries, regions and medical centres to conduct multicentre, large-sample prospective studies to obtain more reliable results. We look forward to further improving the predictive power in future studies by applying more advanced and reliable computer technology.

## Conclusion

We built six machine learning models, decision tree, random forest, gradient boosting machine, support vector machine, neural network, and regularized discriminant analysis, and compared them with the logistic regression model. Tenfold cross-validation was applied to each model, and the final comparison of the AUC values calculated by the confusion matrix revealed that all machine learning models, except decision trees, performed more accurately than logistic regression. Thus, in general, machine learning performed better than logistic regression in predicting new fractures after OVCF, with random forest having the highest accuracy.

## Data Availability

The original data in the study will be made available by the authors, and further inquiries can be directed to the corresponding author.
